# Supplementary material for: Childhood and Adolescent Central Nervous System Tumours in Spain: Incidence and Survival over 20 Years: A Historical Baseline for Current Assessment
Source: Cancers (Basel). 2023 Dec 18;15(24):5889. doi: 10.3390/cancers15245889 (PMC10742240; doi:10.3390/cancers15245889)
Supplement: Supplementary file 1 [file cancers-15-05889-s001.zip › Supplementary Material.pdf]

Supplementary material

INCIDENCE

Table S1. Number of cases of CNS tumours in children and adolescents, according to the International Classification of Childhood Cancer, 3rd Edition (ICCC-3), in Spain, 1983-2007, by incidence and survival period, and their respective percentages. All percentages are with respect to total tumours in **III.** Central Nervous System.

Morphology ICD-O-3		Incidence period (1983-2007)				Survival period (1991-2005)			
		0-14 years		15-19 years		0-14 years		15-19 years	
		N	%	N	%	N	%	N	%
**III CENTRAL NERVOUS SYSTEM**		**1,635**	**100.0**	**328**	**100.0**	**972**	**100.0**	**199**	**100.0**
**IIIa. EPENDYMOMAS AND CHOROID PLEXUS TUMOURS**		**163**	**10.0**	**20**	**6.1**	**84**	**8.6**	**16**	**8.0**
**IIIa1. Ependymomas**		**137**	**8.4**	**20**	**6.1**	**75**	**7.7**	**16**	**8.0**
93831	Subependymoma	1	0.1	2	0.6	0	0.0	2	1.0
93913	Ependymoma, NOS	91	5.6	13	4.0	48	4.9	10	5.0
93923	Ependymoma, anaplastic	32	2.0	2	0.6	19	2.0	2	1.0
93933	Papillary ependymoma	1	0.1	1	0.3	1	0.1	0	0.0
93941	Myxopapillary ependymoma	12	0.7	2	0.6	7	0.7	2	1.0
**IIIa2. Choroid plexus tumour**		**26**	**1.6**	**0**	**0.0**	**9**	**0.9**	**0**	**0.0**
93900	Choroid plexus papilloma, NOS	11	0.7	0	0.0	4	0.4	0	0.0
93901	Atypical choroid plexus papilloma	1	0.1	0	0.0	0	0.0	0	0.0
93903	Choroid plexus carcinoma	14	0.9	0	0.0	5	0.5	0	0.0
**IIIb. ASTROCYTOMAS**		**682**	**41.7**	**145**	**44.2**	**416**	**42.8**	**98**	**49.2**
93803	Glioma, malignant (topo 72.3)	39	2.4	1	0.3	22	2.3	1	0.5
93841	Subependymal giant cell astrocytoma	7	0.4	4	1.2	6	0.6	3	1.5
94003	Astrocytoma, NOS	371	22.7	74	22.6	212	21.8	45	22.6
94013	Astrocytoma, anaplastic	21	1.3	5	1.5	14	1.4	3	1.5
94103	Protoplasmic astrocytoma	13	0.8	4	1.2	10	1.0	1	0.5
94113	Gemistocytic astrocytoma	0	0.0	1	0.3	0	0.0	1	0.5
94203	Fibrillary astrocytoma	21	1.3	4	1.2	16	1.6	3	1.5
94211	Pilocytic astrocytoma	169	10.3	30	9.1	113	11.6	25	12.6
94233	Polar spongioblastoma	2	0.1	0	0.0	0	0.0	0	0.0
94243	Pleomorphic xanthoastrocytoma	8	0.5	2	0.6	7	0.7	2	1.0
94403	Glioblastoma, NOS	28	1.7	17	5.2	13	1.3	12	6.0
94413	Giant cell glioblastoma	1	0.1	2	0.6	1	0.1	1	0.5
94421	Gliofibroma	0	0.0	0	0.0	0	0.0	0	0.0
94423	Gliosarcoma	2	0.1	1	0.3	2	0.2	1	0.5
**IIIc. INTRACRANIAL AND INTRASPINAL EMBRYONAL TUMOURS**		**345**	**21.1**	**40**	**12.2**	**201**	**20.7**	**22**	**11.1**
**IIIc1. Medulloblastomas**		**291**	**17.8**	**32**	**9.8**	**160**	**16.5**	**14**	**7.0**
94703	Medulloblastoma, NOS	270	16.5	28	8.5	148	15.2	12	6.0
94713	Desmoplastic nodular medulloblastoma	16	1.0	4	1.2	8	0.8	2	1.0
94723	Medullomyoblastoma	2	0.1	0	0.0	1	0.1	0	0.0
94743	Large cell medulloblastoma	3	0.2	0	0.0	3	0.3	0	0.0
94803	Cerebellar sarcoma, NOS	0	0.0	0	0.0	0	0.0	0	0.0
**IIIc2. PNET**		**44**	**2.7**	**8**	**2.4**	**36**	**3.7**	**8**	**4.0**
94733	Primitive neuroectodermal tumour, NOS	44	2.7	8	2.4	36	3.7	8	4.0
**IIIc3. Medulloepithelioma**		**5**	**0.3**	**0**	**0.0**	**2**	**0.2**	**0**	**0.0**
95010	Medulloepithelioma, benign	0	0.0	0	0.0	0	0.0	0	0.0
95013	Medulloepithelioma, NOS	2	0.1	0	0.0	0	0.0	0	0.0
95020	Teratoid medulloepithelioma, benign	0	0.0	0	0.0	0	0.0	0	0.0
95023	Teratoid medulloepithelioma	0	0.0	0	0.0	0	0.0	0	0.0
95033	Neuroepithelioma, NOS	3	0.2	0	0.0	2	0.2	0	0.0
95043	Spongioneuroblastoma	0	0.0	0	0.0	0	0.0	0	0.0
**IIIc4. Atypical teratoid/rhabdoid tumour**		**5**	**0.3**	**0**	**0.0**	**3**	**0.3**	**0**	**0.0**
95083	Atypical teratoid/rhabdoid tumour	5	0.3	0	0.0	3	0.3	0	0.0
**IIId. OTHER GLIOMAS**		**170**	**10.4**	**26**	**7.9**	**98**	**10.1**	**11**	**5.5**
**IIId1. Oligodendrogliomas**		**30**	**1.8**	**10**	**3.0**	**12**	**1.2**	**2**	**1.0**
94503	Oligodendroglioma, NOS	28	1.7	9	2.7	10	1.0	2	1.0
94513	Oligodendroglioma, anaplastic	1	0.1	1	0.3	1	0.1	0	0.0
94603	Oligodendroblastoma	1	0.1	0	0.0	1	0.1	0	0.0
**IIId2. Mixed and unspecified gliomas**		**134**	**8.2**	**15**	**4.6**	**81**	**8.3**	**8**	**4.0**
93803	Glioma, malignant	128	7.8	12	3.7	79	8.1	7	3.5
93823	Mixed glioma	6	0.4	3	0.9	2	0.2	1	0.5
**IIId3. Neuroepithelial glial tumours of uncertain origin**		**6**	**0.4**	**1**	**0.3**	**5**	**0.5**	**1**	**0.5**
93813	Gliomatosis cerebri	4	0.2	0	0.0	3	0.3	0	0.0
94303	Astroblastoma	2	0.1	1	0.3	2	0.2	1	0.5
94441	Chordoid glioma	0	0.0	0	0.0	0	0.0	0	0.0

Morphology ICD-O-3		Incidence period (1983-2007)				Survival period (1991-2005)			
		0-14 years		15-19 years		0-14 years		15-19 years	
		N	%	N	%	N	%	N	%
IIIe. OTHER SPECIFIED INTRACRANIAL AND INTRASPINAL NEOPLASMS		136	8.3	52	15.9	88	9.1	27	13.6
**IIIe1. Pituitary adenomas and carcinomas**		**13**	**0.8**	**11**	**3.4**	**6**	**0.6**	**6**	**3.0**
82700	Chromophobe adenoma	3	0.2	1	0.3	1	0.1	0	0.0
82703	Chromophobe carcinoma	0	0.0	0	0.0	0	0.0	0	0.0
82710	Prolactinoma	5	0.3	6	1.8	3	0.3	5	2.5
82720	Pituitary adenoma, NOS	3	0.2	4	1.2	1	0.1	1	0.5
82723	Pituitary carcinoma, NOS	0	0.0	0	0.0	0	0.0	0	0.0
82800	Acidophil adenoma	1	0.1	0	0.0	0	0.0	0	0.0
82803	Acidophil carcinoma	0	0.0	0	0.0	0	0.0	0	0.0
82810	Mixed acidophil-basophil adenoma	0	0.0	0	0.0	0	0.0	0	0.0
82813	Mixed acidophil-basophil carcinoma	0	0.0	0	0.0	0	0.0	0	0.0
83000	Basophil adenoma	1	0.1	0	0.0	1	0.1	0	0.0
83003	Basophil carcinoma	0	0.0	0	0.0	0	0.0	0	0.0
**IIIe2. Tumours of the sellar region (craniopharyngiomas)**		**64**	**3.9**	**9**	**2.7**	**42**	**4.3**	**5**	**2.5**
93501	Craniopharyngioma	63	3.9	9	2.7	41	4.2	5	2.5
93511	Craniopharyngioma, adamantinomatous	1	0.1	0	0.0	1	0.1	0	0.0
93521	Craniopharyngioma, papillary	0	0.0	0	0.0	0	0.0	0	0.0
95820	Granular cell tumour of sellar region	0	0.0	0	0.0	0	0.0	0	0.0
**IIIe3. Pineal parenchymal tumours**		**17**	**1.0**	**5**	**1.5**	**9**	**0.9**	**1**	**0.5**
93601	Pinealoma	6	0.4	2	0.6	4	0.4	0	0.0
93611	Pineocytoma	1	0.1	1	0.3	0	0.0	1	0.5
93623	Pineoblastoma	10	0.6	2	0.6	5	0.5	0	0.0
**IIIe4. Neuronal and mixed neuronal-glial tumours**		**19**	**1.2**	**5**	**1.5**	**14**	**1.4**	**4**	**2.0**
94121	Desmoplastic infantile astrocytoma	1	0.1	0	0.0	1	0.1	0	0.0
94130	Dysembryoplastic neuroepithelial tumour	4	0.2	1	0.3	3	0.3	0	0.0
94920	Gangliocytoma	1	0.1	0	0.0	1	0.1	0	0.0
94930	Dysplastic gangliocytoma of cerebellum (Lhermitte-Duclos)	0	0.0	0	0.0	0	0.0	0	0.0
95051	Ganglioglioma, NOS	12	0.7	1	0.3	9	0.9	1	0.5
95053	Ganglioglioma, anaplastic	0	0.0	1	0.3	0	0.0	1	0.5
95061	Central neurocytoma	1	0.1	2	0.6	0	0.0	2	1.0
95070	Pacinian tumour	0	0.0	0	0.0	0	0.0	0	0.0
**IIIe5. Meningiomas**		**23**	**1.4**	**12**	**3.7**	**17**	**1.7**	**11**	**5.5**
95300	Meningioma, NOS	6	0.4	7	2.1	3	0.3	6	3.0
95301	Meningiomatosis, NOS	2	0.1	0	0.0	2	0.2	0	0.0
95303	Meningioma, malignant	7	0.4	3	0.9	6	0.6	3	1.5
95310	Meningothelial meningioma	2	0.1	0	0.0	2	0.2	0	0.0
95320	Fibrous meningioma	3	0.2	1	0.3	1	0.1	1	0.5
95330	Psammomatous meningioma	1	0.1	0	0.0	1	0.1	0	0.0
95340	Angiomatous meningioma	0	0.0	0	0.0	0	0.0	0	0.0
95350	Hemangioblastic meningioma	0	0.0	0	0.0	0	0.0	0	0.0
95370	Transitional meningioma	0	0.0	1	0.3	0	0.0	1	0.5
95381	Clear cell meningioma	1	0.1	0	0.0	1	0.1	0	0.0
95383	Papillary meningioma	1	0.1	0	0.0	1	0.1	0	0.0
95391	Atypical meningioma	0	0.0	0	0.0	0	0.0	0	0.0
95393	Meningeal sarcomatosis	0	0.0	0	0.0	0	0.0	0	0.0
**IIIe6. Others**		**0**	**0.0**	**10**	**3.0**	**0**	**0.0**	**0**	**0.0**
87281	Meningeal melanocytoma	0	0.0	0	0.0	0	0.0	0	0.0
88500	Lipoma, NOS	0	0.0	0	0.0	0	0.0	0	0.0
91210	Cavernous hemangioma	0	0.0	1	0.3	0	0.0	0	0.0
91611	Hemangioblastoma	0	0.0	3	0.9	0	0.0	0	0.0
91730	Cystic lymphangioma	0	0.0	1	0.3	0	0.0	0	0.0
93630	Melanotic neuroectodermal tumor	0	0.0	0	0.0	0	0.0	0	0.0
95600	Neurilemoma, NOS	0	0.0	5	1.5	0	0.0	0	0.0
IIIf. UNSPECIFIED INTRACRANIAL AND INTRASPINAL NEOPLASMS		139	8.5	45	13.7	85	8.7	25	12.6
80000	Neoplasm, benign	11	0.7	1	0.3	9	0.9	1	0.5
80001	Neoplasm, uncertain whether benign or malignant	29	1.8	8	2.4	19	2.0	3	1.5
80003	Neoplasm, malignant	94	5.7	34	10.4	54	5.6	20	10.1
80010	Tumour cells, benign	0	0.0	0	0.0	0	0.0	0	0.0
80011	Tumour cells, uncertain whether benign or malignant	0	0.0	0	0.0	0	0.0	0	0.0
80013	Tumour cells, malignant	1	0.1	1	0.3	1	0.1	1	0.5
80023	Malignant tumour, small cell type	4	0.2	1	0.3	2	0.2	0	0.0
80033	Malignant tumour, giant cell type	0	0.0	0	0.0	0	0.0	0	0.0
80043	Malignant tumour, spindle cell type	0	0.0	0	0.0	0	0.0	0	0.0
80050	Clear cell tumour, NOS	0	0.0	0	0.0	0	0.0	0	0.0
80053	Malignant tumour, clear cell type	0	0.0	0	0.0	0	0.0	0	0.0

Children: patients aged 0-14 year; Adolescents: patients aged 15-19 year; CNS: central nervous system (Group III of the ICCC-3); ICCC-3: International Classification of Childhood Cancer 3rd ed. [25]

Table S2. Number of cases of central nervous system (CNS) tumours according to the ICCC-3, by sex and total, and age groups: period 1983-2007.

ICCC-3 CNS group	N Boys					N Girls					N All				
	0-4	5-9	10-14	0-14	15-19	0-4	5-9	10-14	0-14	15-19	0-4	5-9	10-14	0-14	15-19
IIIa. Ependymomas and choroid plexus tumors	50	29	26	105	13	31	15	12	58	7	81	44	38	163	20
IIIb. Astrocytomas	125	111	119	355	81	106	110	111	327	64	231	221	230	682	145
IIIc. Intracranial and intraspinal embryonal tumors	84	85	46	215	24	49	50	31	130	16	133	135	77	345	40
IIId. Other gliomas	35	32	25	92	14	26	27	25	78	12	61	59	50	170	26
IIIe. Other specified intracranial and intraspinal neoplasms	14	28	37	79	24	11	18	28	57	28	25	46	65	136	52
IIIf. Unspecified intracranial and intraspinal neoplasms	19	20	28	67	24	21	30	21	72	21	40	50	49	139	45
III. All CNS	327	305	281	913	180	244	250	228	722	148	571	555	509	1635	328

CNS: central nervous system (Group III of the ICCC-3); ICCC-3: International Classification of Childhood Cancer 3rd ed. [25]; N: number of cases.

Table S3. Age-specific incidence rates of central nervous system (CNS) tumours (IR) per million,[1] according to the ICCC-3, by sex and total: period 1983-2007.

ICCC-3 CNS group	IR Boys					IR Girls					IR All				
	0-4	5-9	10-14	0-14	15-19	0-4	5-9	10-14	0-14	15-19	0-4	5-9	10-14	0-14	15-19
IIIa. Ependymomas and choroid plexus tumours	6.3	3.3	2.6	4.0	1.8	4.2	1.8	1.3	2.3	1.0	5.3	2.6	2.0	3.2	1.4
IIIb. Astrocytomas	15.8	12.8	12.1	13.4	11.3	14.2	13.4	11.9	13.1	9.4	15.0	13.1	12.0	13.3	10.4
IIIc. Intracranial and intraspinal embryonal tumours	10.6	9.8	4.7	8.1	3.4	6.6	6.1	3.3	5.2	2.3	8.6	8.0	4.0	6.7	2.9
IIId. Other gliomas	4.4	3.7	2.5	3.5	2.0	3.5	3.3	2.7	3.1	1.8	4.0	3.5	2.6	3.3	1.9
IIIe. Other specified intracranial and intraspinal neoplasms	1.8	3.2	3.8	3.0	3.4	1.5	2.2	3.0	2.3	4.1	1.6	2.7	3.4	2.6	3.7
IIIf. Unspecified intracranial and intraspinal neoplasms	2.4	2.3	2.8	2.5	3.4	2.8	3.7	2.2	2.9	3.1	2.6	3.0	2.6	2.7	3.2
III. All CNS	41.3	35.2	28.6	34.5	25.2	32.7	30.4	24.4	28.9	21.7	37.1	32.9	26.5	31.8	23.5

[1] (Boys/girls/all)
CNS: central nervous system (Group III of the ICCC-3); ICCC-3: International Classification of Childhood Cancer 3rd ed. [25]; IR: age-specific incidence rates.

Table S4. Age-standardised incidence rates, world standard population (ASRw), per million[1] of central nervous system (CNS) tumours according to the ICCC-3, by sex and total, and age group 0-14 and 0-19 year: period 1983-2007.

ICCC-3 CNS group	ASRw Boys		ASRw Girls		ASRw All	
	0-14	0-19	0-14	0-19	0-14	0-19
IIIa. Ependymomas and choroid plexus tumours	4.3	3.7	2.6	2.2	3.5	3.0
IIIb. Astrocytomas	13.7	13.2	13.3	12.4	13.5	12.8
IIIc. Intracranial and intraspinal embryonal tumours	8.6	7.4	5.5	4.8	7.1	6.1
IIId. Other gliomas	3.6	3.3	3.2	2.9	3.4	3.1
IIIe. Other specified intracranial and intraspinal neoplasm	2.8	2.9	2.1	2.6	2.5	2.8
IIIf. Unspecified intracranial and intraspinal neoplasms	2.5	2.7	2.9	3.0	2.7	2.8
III. All CNS	35.6	33.3	29.5	27.8	32.7	30.6

[1] (Boys/girls/all)
CNS: central nervous system (Group III of the ICCC-3); ICCC-3: International Classification of Childhood Cancer 3rd ed. [25]; ASRw: age-standardised incidence rates, world standard population.

SURVIVAL

-Note: In the following tables survival results are not shown for <10 cases, log rank results are not shown when there are no survival results for all age groups, and log rank for trend results by age group, as well as annual percent change, when there are no survival results for the three cohorts.

Table S5. CNS tumours (malignant and non-malignant combined) in children, total and by diagnostic subgroups, period 1991-2005, by cohort of diagnosis. 5-year observed survival by age group, and standardised survival. Log rank comparing the equality of survival distributions by age group, log rank trend for observed survival, annual percent change for standardised survival and 5-year follow-up for the 0-14 year age group.

	Cohort of diagnosis						
	1991-1995		1996-2000		2001-2005		
Age group	n	5-y observed survival (%) (95% Ci)	n	5-y observed survival (%) (95% CI)	n	5-y observed survival (%) (95% CI)	Log rank trend *(p)*
**III. All CNS tumours**							
0	21	43(22;64)	13	77(54;100)	15	60(35;85)	0.194
1-4	108	59(49;68)	97	66(57;75)	103	57(48;67)	0.502
5-9	102	57(47;67)	107	58(48;67)	107	63(53;72)	0.611
10-14	118	75(67;83)	94	77(68;85)	87	84(76;92)	0.298
0-14	349	63(58;68)	311	67(62;72)	312	67(61;72)	0.260
Log rank(*p*)		0.002		0.055		0.001	
Standardised survival 0-14 years		62(57;67)		67(62;72)		67(62;72)	APC (95% CI) 4(-19;34)
**IIIa-Ependymomas and choroid plexus tumours**							
0	5		0		4		
1-4	12	33(7;60)	8		16	31(9;54)	
5-9	8		4		8		
10-14	8		7		4		
0-14	33	46(28;63)	19	58(36;80)	32	47(30;64)	0.824
Standardised survival 0-14 years		45(29;61)		52(31;74)		54(40;67)	APC (95% CI) 8(-26;58)
**IIIb-Astrocytomas**							
0	9		6		7		
1-4	48	83(72;94)	43	86(76;96)	38	95(88;100*)	0.260
5-9	40	75(62;88)	51	77(65;88)	36	81(68;94)	0.873
10-14	48	87(78;97)	48	79(68;91)	42	90(81;99)	0.277
0-14	145	80(73;86)	148	81(75;87)	123	89(83;94)	0.067
Standardised survival 0-14 years		80(73;86)		82(75;88)		88(83;94)	APC (95% CI) 6(-15;31)

Table S5. (contin.)

Age group	1991-1995 n	1991-1995 5-y observed survival (%) (95% CI)	1996-2000 n	1996-2000 5-y observed survival (%) (95% CI)	2001-2005 n	2001-2005 5-y observed survival (%) (95% CI)	Log rank trend *(p)*
	Cohort of diagnosis						
**IIIc-Intracranial and intraspinal embryonal tumours**							
0	3		3		2		0.136
1-4	25	28(10;46)	24	46(26;66)	26	35(16;53)	0.619
5-9	25	52(32;72)	22	41(20;61)	26	54(35;73)	0.676
10-14	19	63(41;85)	12	75(51;100)	14	71(48;95)	0.878
0-14	72	46(34;57)	61	51(38;63)	68	49(37;60)	0.995
Standardised survival 0-14 years		45(34;56)		51(39;63)		48(37;60)	APC (95% CI) 4(-49;112)
**IIId-Other gliomas**							
0	1		1		1		
1-4	12	58(30;86)	11	64(35;92)	9		
5-9	11	36(8;65)	10	50(19;81)	15	27(4;49)	0.619
10-14	14	57(31;83)	8		5		
0-14y	38	50(34;66)	30	53(35;71)	30	30(14;46)	0.189
Standardised survival 0-14 years		48(33;64)		53(35;70)		35(22;48)	APC (95% CI) -14(-87;455)
**IIIe-Other specified intracranial and intraspinal neoplasms**							
0	1		0		1		
1-4	3		5		8		
5-9	9		11	61(30;91)	9		
10-14	12	100	13	77(54;100)	16	94(82;100*)	0.122
0-14	25	80(64;96)	29	68(51;85)	34	85(73;97)	0.506
Standardised survival 0-14 years		79(65;94)		68(51;85)		84(72;96)	APC (95% CI) 4(-60;170)
**IIIf-Unspecified intracranial and intraspinal neoplasms**							
0	2		3		0		
1-4	8		6		6		
5-9	9		9		13	69(44;94)	
10-14	17	53(29;77)	6		6		
0-14	36	47(31;63)	24	42(22;61)	25	52(32;72)	0.492
Standardised survival 0-14 years		44(29;59)		48(36;60)		47(28;66)	APC (95% CI) 4(-37;72)

CNS: central nervous system (Group III of the ICCC-3); ICCC-3: International Classification of Childhood Cancer 3rd ed. [25]; n: number of cases; 5-y: 5-year survival; (95% CI): confidence interval; APC: annual percent change. *****Truncated upper limit.

Table S6. CNS tumours (malignant and non-malignant combined) in adolescents by diagnostic subgroups of the ICCC-3, period 1991-2005, by cohort of diagnosis. 5-year observed survival and follow up. Log rank trend for observed survival.

See note at top of first page of Supplementary Survival Tables

Cohort of diagnosis						
1991-1995		1996-2000		2001-2005		
n	5-y observed survival (%) (95% CI)	n	5-y observed survival (%) (95% CI)	n	5-y observed survival (%) (95% CI)	Log rank trend *(p)*
III. All CNS tumours						
67	66(54;77)	69	71(60;81)	63	58(46;70)	0.380
IIIb-Astrocytomas						
40	73(59;86)	30	76(61;91)	28	64(46;82)	0.650
IIIe-Other specified						
5		13	92(78;100*)	9		

CNS: central nervous system (Group III of the ICCC-3); ICCC-3: International Classification of Childhood Cancer 3rd ed. [25]; n: number of cases; 5-y: 5-year survival; (95% CI): confidence interval.

Table S7. Pilocytic astrocytoma[a] in children, period 1991-2005. 5-year observed survival by cohort of diagnosis and age group, and standardised survival. Log rank comparing the equality of survival distributions by age group, log rank trend for observed survival, annual percent change for standardised survival, and 5-year follow up for the 0-14 year age group.

See note at the top of first page of Supplementary Survival Tables

	Cohort of diagnosis						
	1991-1995		1996-2000		2001-2005		
Age group	n	5-y observed survival (%) (95% CI)	n	5-y observed survival (%) (95% CI)	n	5-y observed survival (%) (95% CI)	Log rank trend *(p)*
0	2		2		2		
1-4	8		14	93(79;100*)	7		
5-9	3		24	88(74;100*)	15	100	
10-14	10	90(71;100*)	12	92(76;100*)	14	100	0.510
0-14	23	91(79;100*)	52	90(82;98)	38	95(88;100*)	0.532
Standardised survival 0-14 years		94(87;100*)		91(83;99)		93(84;100*)	APC (95% CI) -1(-19;21)

n: number of cases; 5-y: 5-year survival; (95% CI): confidence interval; APC: annual percent change; [a] ICD-O-3 morphology code: 9421/1; ICD-O-3: International Classification of Diseases for Oncology 3rd ed. [26]; *Truncated upper limit.

Table S8. Astrocytoma NOS[a] in children, period 1991-2005. 5-year observed survival by cohort of diagnosis and age group, and standardised survival. Log rank comparing the equality of survival distributions by age group, log rank trend for observed survival, annual percent change for standardised survival, and 5-year follow up for the 0-14 year age group.

	Cohort of diagnosis						
	1991-1995		1996-2000		2001-2005		
Age group	n	5-y observed survival (%) (95% CI)	n	5-y observed survival (%) (95% CI)	n	5-y observed survival (%) (95% CI)	Log rank trend *(p)*
0	5		4		3		
1-4	32	78(64;92)	21	91(78;100*)	18	100	0.083
5-9	33	76(61;91)	16	81(62;100*)	13	77(54;100)	0.951
10-14	31	87(75;99)	24	71(53;89)	12	92(76;100*)	0.197
0-14	101	79(71;87)	65	82(72;91)	46	89(80;98)	0.208
Standardised survival 0-14 years		79(71;87)		82(73;91)		89(80;98)	APC (95% CI) 6(-8;22)

n: number of cases; 5-y: 5-year survival; (95% CI): confidence interval; APC: annual percent change; NOS: not otherwise specified; [a] ICD-O-3 morphology code: 9400/3; ICD-O-3: International Classification of Diseases for Oncology 3rd ed. [26]; *Truncated upper limit; ^Statistically significant trend.

Table S9 Malignant[a] and non-malignant[b] CNS tumours in children, period 1991-2005, by cohort of diagnosis and behaviour. 5-year observed survival by age group, and standardised survival. Log rank comparing the equality of survival distributions by age group, log rank trend for observed survival, annual percent change for standardised survival, and 5-year follow up for the 0-14 year age group.

See note at top of first page of Supplementary Survival Tables

Age group	Cohort of diagnosis 1991-1995 n	5-y observed survival (%) (95% CI)	1996-2000 n	5-y observed survival (%) (95% CI)	2001-2005 n	5-y observed survival (%) (95% CI)	Log rank trend *(p)*
Malignant tumours							
0	17	35(13;58)	10	70(42;98)	11	46(16;75)	0.286
1-4	94	53(43;63)	76	61(50;71)	86	54(43;64)	0.667
5-9	88	57(46;67)	71	47(35;58)	77	49(38;61)	0.436
10-14	94	70(60;79)	64	70(59;81)	50	80(69;91)	0.411
0-14	293	58(53;64)	221	59(53;66)	224	58(51;64)	0.879
Log Rank *(p)*		0.005		0.073		0.006	
Standardised survival 0-14 years		58(52;64)		59(53;66)		59(53;65)	APC (95% CI) 1(-8;11)
Non-malignant tumours							
0	4		3		4		
1-4	14	100	21	86(71;100*)	17	77(56;97)	0.185
5-9	14	57(31;83)	36	80(67;93)	30	97(90;100*)	0.003
10-14	24	96(88;100*)	30	90(79;100*)	37	89(79;99)	0.668
0-14	56	86(76;95)	90	85(78;93)	88	90(84;96)	0.390
Standardised survival 0-14 years		83(73;92)		86(79;93)		89(83;96)	APC (95% CI) 4^(2;6)

CNS: central nervous system (Group III of the ICCC-3); ICCC-3: International Classification of Childhood Cancer 3rd ed. [25]; n: number of cases; 5-y: 5-year survival; (95% CI): confidence interval; APC: annual percent change; [a] Malignant: 5th digit of the morphology code of the ICD-O-3 equal 3; [b] Non-malignant: 5th digit of the morphology code of the ICD-O-3 < 3; ICD-O-3: International Classification of Diseases for Oncology 3rd ed. [26]; *Truncated upper limit; ^Statistically significant trend.

Table S10. Lethal CNS tumours[a] in children, period 1991-2005, by cohort of diagnosis. 5-year observed survival by age group, and standardised survival. Log rank comparing the equality of survival distributions by age groups, log rank trend for observed survival, annual percent change for standardised survival, and 5-year follow up for the 0-14 year age group.

See note at top of first page of Supplementary Survival Tables

	Cohort of diagnosis					
	1991-1995		1996-2000		2001-2005	
Age group	n	5-y observed survival (%) (95% CI)	n	5-y observed survival (%) (95% CI)	n	5-y observed survival (%) (95% CI)
0-14	10	33(3;64)	9		15	53(27;78)
Standardised survival 0-14 years		32(9;54)				49(26;73)

CNS: central nervous system (Group III of the ICCC-3); ICCC-3: International Classification of Childhood Cancer 3rd ed. [25]; n: number of cases; 5-y: 5-year survival; (95% CI): confidence interval; APC: annual percent change; [a] ICD-O-3 morphology codes: 9508/3, 9401/3, 9451/3, 9440/3, 9441/3, 9442/3; ICD-O-3: International Classification of Diseases for Oncology 3rd ed. [26].

Table S11. Gliomas[a] in children, period 1991-2005, by cohort of diagnosis. 5-year observed survival by age group, and standardised survival. Log rank comparing the equality of survival distributions by age groups and log rank trend for observed survival, annual percent change for standardised survival, and 5-year follow up for the 0-14 year age group.

See note at top of first page of Supplementary Survival Tables

	Cohort of diagnosis						
	1991-1995		1996-2000		2001-2005		
Age group	n	5-y observed survival (%) (95% CI)	n	5-y observed survival (%) (95% CI)	n	5-y observed survival (%) (95% CI)	Log rank trend *(p)*
0-14	28	43(24;61)	23	48(27;68)	28	25(9;41)	0.342
							APC (95% CI)
Standardised survival 0-14 years		41(23;60)		46(27;65)		31(17;44)	-14(-89;581)

n: number of cases; 5-y: 5-year survival; (95% CI): confidence interval; APC: annual percent change; [a] ICD-O-3 morphology code: 9380/3, optical tract excluded; ICD-O-3: International Classification of Diseases for Oncology 3rd ed. [26].

Table-S12. CNS tumours in children in Spain (2001-05) and Europe (2000-07). Relative frequency distribution by diagnostic subgroups of Group III of ICCC-3.

Diagnostic subgroup	WHO grade	Europe 2000-2007		Spain 2001-2005		
		N	%	N	%	*p*
**IIIa-Ependymoma and choroid plexus**		**1,534**	**10.2**	**32**	**10.3**	
Choroid plexus papilloma	I	172	11.2	2	6.3	
Subependymoma	I	20	1.3	0	0.0	
Myxopapillary ependymoma	I	75	4.9	4	12.5	
Atypical choroid plexus papilloma	II	35	2.3	0	0.0	
Ependymoma, other and NOS [a]	II	591	38.5	14	43.8	
Choroid plexus carcinoma	III	150	9.8	1	3.1	
Anaplastic ependymoma	III	491	32.0	11	34.4	
**IIIb-Astrocytomas**		**6,078**	**40.5**	**123**	**39.4**	
Pilocytic astrocytoma	I	3,231	53.2	38	30.9	*
Subependymal giant cell astrocytoma	I	136	2.2	3	2.4	
Glioma (optic nerve)	I	611	10.1	13	10.6	
Pleomorphic xanthoastrocytoma	II	73	1.2	3	2.4	
Fibrillary astrocytoma	II	182	3.0	5	4.1	
Protoplasmic astrocytoma	II	19	0.3	3	2.4	*
Gemistocytic astrocytoma	II	8	0.1	0	0.0	
Anaplastic astrocytoma	III	338	5.6	5	4.1	
Glioblastoma and variants [b]	IV	530	8.7	7	5.7	
Astrocytomas NOS [c]	-	948	15.6	46	37.4	*
Gliofibroma	-	2	0.0	0	0.0	
**IIIc-Intracranial and intraspinal embryonal tumours**		**3,097**	**20.6**	**68**	**21.8**	
Medulloblastoma, variants [d]	IV	2,006	64.8	47	69.1	
Medulloblastoma large cell	IV	52	1.7	1	1.5	
Desmoplastic/nodular medulloblastoma	IV	237	7.7	3	4.4	
PNET, variants	IV	544	17.6	14	20.6	
Atypical teratoid/rhabdoid tumour	IV	258	8.3	3	4.4	
**IIId-Other gliomas**		**1,642**	**10.9**	**30**	**9.6**	
Oligodendroglioma [e]	II	212	12.9	1	3.3	
Oligodendroglioma, anaplastic	III	91	5.5	0	0.0	
Glioma, mixed	III	137	8.3	1	3.3	
Astroblastoma	-	19	1.2	0	0.0	
Chordoid glioma	-	1	0.1	0	0.0	
Gliomatosi cerebri	-	30	1.8	0	0.0	
Glioma NOS (excluding optic nerve)	-	1,152	70.2	28	93.3	

Table S12 continues on next page

Table-S12. (contin.)

Diagnostic subgroup	WHO grade	Europe 2000-2007		Spain 2001-2005		
		N	%	N	%	*p*
**IIIe-Other specified CNS tumours**		**1,866**	**12.4**	**34**	**10.9**	
Pinealoma and pineocytoma	I	19	1.0	1	2.9	
Desmoplastic infantile astrocytoma	I	59	3.2	1	2.9	
Dysembryoplastic neuroepithelial tumour	I	322	17.3	2	5.9	
Gangliocytomas, ganglioglioma [f]	I	402	21.5	5	14.7	
Meningioma, non-malignant [g]	I	188	10.1	3	8.8	
Craniopharyngioma[h]	I	608	32.6	17	50.0	
Central neurocytoma	II	17	0.9	0	0.0	
Ganglioglioma, anaplastic	III	25	1.3	0	0.0	
Meningioma, malignant [i]	III	29	1.6	2	5.9	
Pineoblastoma	IV	105	5.6	2	5.9	
Pituitary tumour [j]	-	92	4.9	1	2.9	
**IIIf-Unspecified CNS**		**800**	**5.3**	**25**	**8.0**	
Malignant	-	429	53.6	14	56.0	
Benign	-	371	46.4	11	44.0	
**III CNS**		**15,017**		**312**		

CNS: central nervous system (Group III of the ICCC-3). ICCC-3: International Classification of Childhood Cancer 3rd ed. [25]. WHO grade[49] as applied by Gatta et al 2017[37]. European data from Gatta et al. 2017[37]. *p*: Adjusted p-value. Chi-squared tests were used to compare between the observed proportions in Spain and those reported in Europe. Yates correction for continuity was used to account for the low frequency of some morphologies. p-values were adjusted for multiple testing using the Benjamini and Hochberg method [50]. *Adjusted p-value < 0.05.

[a] Includes ependymoma, NOS and papillary ependymoma. [b] Includes glioblastoma, NOS; giant cell glioblastoma and gliosarcoma. [c] Includes astrocytoma, NOS and polar spongioblastoma. [d] Includes medulloblastoma, NOS; medullomyoblastoma; cerebellar sarcoma, NOS; medulloepithelioma, NOS and neuroepithelioma, NOS. [e] Includes oligodendroglioma, NOS and oligodendroblastoma. [f] Includes gangliocytoma; dysplastic gangliocytoma of cerebellum (Lhermitte-Duclos) and ganglioglioma, NOS.
[g]Includes meningioma, NOS; meningiomatosis, NOS; meningothelial meningioma; fibrous meningioma; psammomatous meningioma; angiomatous meningioma; hemangioblastic meningioma; transitional meningioma; clear cell meningioma and atypical meningioma. [h] Includes craniopharyngioma, adamantinomatous craniopharyngioma and papillary craniopharyngioma. [I] Includes meningioma, malignant; papillary meningioma and meningeal sarcomatosis. [j] Includes chromophobe adenoma; prolactinoma; pituitary adenoma, NOS; acidophil adenoma; mixed acidophil-basophil adenoma and basophil adenoma.

Table S13. Percentage of non-malignant tumours in children registered across the study period (incidence period 1983-2007) by individual registry and five-year period.

	Incidence period					
**Registry**	1983-1987	1988-1992	1993-1997	1998-2002	2003-2007	**Total**
Albacete	0.0	0.0	0.0	0.0	0.0	0.0
Asturias	4.5	12.5	30.8	30.8	25.0	18.5
Balearic Is	0.0	10.5	18.8	41.2	30.0	24.2
Basque Country	19.2	22.9	32.4	50.0	27.6	30.3
Girona	11.8	27.8	22.2	28.6	46.7	27.0
Granada	14.3	20.0	23.5	35.3	31.6	25.3
Murcia	0.0	0.0	0.0	0.0	0.0	0.0
Navarre	25.0	0.0	27.3	22.7	21.4	21.0
Tarragona	8.3	8.3	28.6	12.5	16.7	15.6
Valencian Community	12.6	12.9	21.5	35.8	30.8	23.1
Zaragoza	25.9	9.1	14.3	25.9	52.4	26.1
**All registries**	13.8	13.7	22.2	31.5	27.9	22.0

Table S14. Percentage of non-malignant tumours in children registered in the survival period (1991-2005) by individual registry[a] and five-year period.

	**Survival period**			
**Registry**	**1991-1995**	**1996-2000**	**2001-2005**	**Total**
Albacete	0.0	0.0	0.0	0.0
Asturias	30.8	21.1	35.3	28.6
Balearic Is	12.5	37.5	35.3	28.6
Basque Country	24.3	47.5	32.6	34.3
Girona	25.0	20.0	44.4	29.8
Granada	8.3	46.2	41.2	27.8
Murcia	0.0	0.0	0.0	0.0
Navarre	10.0	26.3	22.2	21.3
Tarragona	0.0	36.4	11.1	17.2
Valencian Community	17.9	25.8	30.7	24.8
**All registries**	16.0	28.9	28.2	24.1

[a] The Zaragoza cancer registry did not contribute to the survival study.

Table S15. Percentage of non-malignant tumours in children registered in 1991-2005 by individual registry. Four registries were excluded due to incomplete registration of non-malignant tumours (<25% of non-malignant tumours).

Registry	Survival period 1991-1995	Survival period 1996-2000	Survival period 2001-2005	Total
Asturias	30.8	21.1	35.3	28.6
Balearic Is	12.5	37.5	35.3	28.6
Basque Country	24.3	47.5	32.6	34.3
Girona	25.0	20.0	44.4	29.8
Granada	8.3	46.2	41.2	27.8
Valencian Community	17.9	25.8	30.7	24.8
**All registries**	19.6	31.9	33.3	27.9

Figure S1. Total percentage of non-malignant tumours registered by all PBCRs across the study period (incidence period 1983-2007), by calendar year.

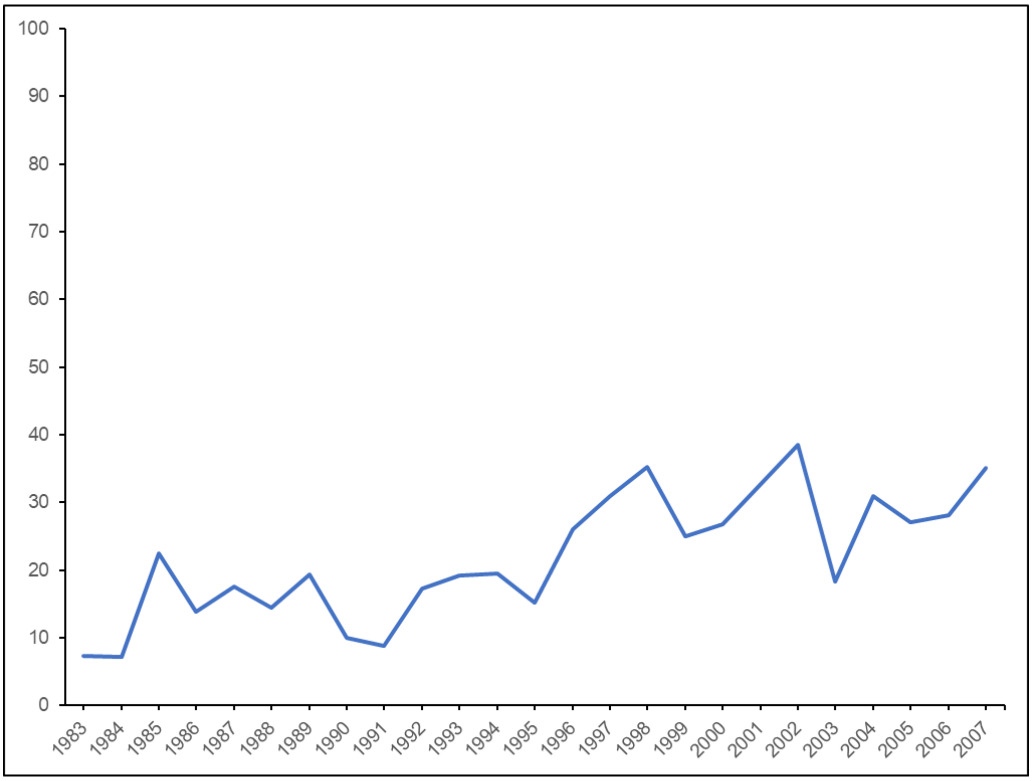

Figure S2. Total percentage of non-malignant tumours registered by the PBCRs participating in the survival period (1991-2005)[a], by calendar year.

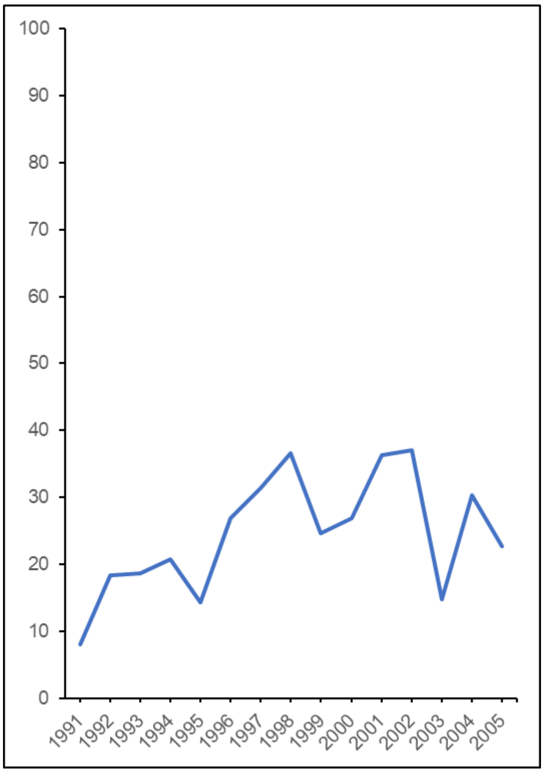

[a] The Zaragoza cancer registry did not contribute to the survival study.
